# The effect of nandrolone decanoate administration on fatigue during a volume‐overload stress in male mice

**DOI:** 10.14814/phy2.70334

**Published:** 2025-05-08

**Authors:** Tavor Ben‐Zeev, David D. Church, Chagai Levi, Inbal Weissman, Abby Fulbright, Avidan Shalev, Ariel Levin, Doron Schussheim, Arny A. Ferrando, Jay R. Hoffman

**Affiliations:** ^1^ School of Health Sciences Ariel University Ariel Israel; ^2^ Center for Translational Research in Aging and Longevity University of Arkansas for Medical Sciences Little Rock Arkansas USA

**Keywords:** anabolic steroids, androgens, exercise, sustained military operations, testosterone

## Abstract

The effect of nandrolone decanoate on fatigue was examined during a volume‐overload training stress in 3‐month‐old male C57Bl/6J mice (*n* = 24). Mice were randomized into two exercising groups and a control group (C). The exercising animals performed a 3‐day per week resistance training program for 3 weeks. Exercising animals were further randomized into an androgen group (RTA) or a sham group (RTS). To exert a volume‐overload, the frequency of training was increased to six consecutive days during week 4. RTA received a supraphysiological dose of nandrolone decanoate (38‐mg·kg^−1^) before and after the volume‐overload week. RTS and C received sham injections. Four mice in RTS were determined to be fatigued, while no mice in RTA were fatigued. TNF‐α expression in the plantaris was significantly lower for RTA compared to RTS. Significant elevations in oxidative stress were noted in RTS compared to C in the plantaris, but no differences were noted between RTA and C, suggesting a lower oxidative stress response from nandrolone decanoate administration. Glucocorticoid expression was significantly lower in the soleus of RTA compared to RTS, suggesting a lower catabolic response to the volume‐overload stress. In conclusion, nandrolone decanoate intervention attenuated fatigue in animals during a volume‐overload stress.

## Graphical abstract

Nandrolone decanoate administrations reduces fatgiue during a volume‐overload stress in mice.

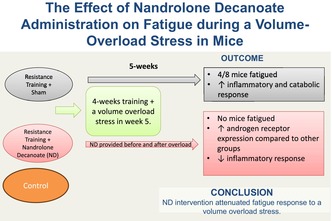

## 1 INTRODUCTION

Highly intense physical activity for a prolonged duration is often required of tactical personnel during training or actual combat missions. These physical demands often occur without adequate recovery and can result in fatigue and decreases in performance (Nindl et al., 2007). Soldiers participating in combat training and/or actual military operations are often carrying heavy loads in difficult environmental conditions (Nindl et al., 2007; Ross & Heebner, 2023; Schuh‐Renner et al., 2017). These stresses are often associated with a reduced caloric intake exacerbating the catabolic effects of such activity (Margolis et al., 2013). Due to the sustained nature of military operations concomitant with limited recovery, soldiers are at an increased risk of experiencing symptoms of fatigue (Jensen et al., 2019; Pasiakos et al., 2015).

Several studies have demonstrated that sustained military training has resulted in a significant decrease in circulating testosterone concentrations (Henning et al., 2014; Jensen et al., 2019; Nindl et al., 1997, 2007). Decrements in testosterone concentrations have been reported to be associated with a significant reduction in body mass and in the cross‐sectional area of both upper and lower body musculature following an 8‐week training operation (Henning et al., 2014; Nindl et al., 2007). In one of these investigations, Henning and colleagues (Henning et al., 2014) reported that a 70% reduction in resting testosterone concentrations required between 2 and 6 weeks of recovery to return to baseline levels. Others have reported that intense military training may also result in significant reductions in whole‐body protein balance (Schuh‐Renner et al., 2017), while other investigations have reported significant reductions in strength and power (Nindl et al., 1997, 2007). The sustained nature of many combat operations may also have a prolonged effect. Jensen et al. (2019) reported that 43% of elite military personnel have chronically low resting testosterone levels, which are often accompanied by elevated cortisol concentrations. These responses resemble what is often observed in the overtrained competitive athlete (Meeusen et al., 2013).

The physiological effect of inadequate recovery following high volume training is similar between the tactical and competitive athlete (Pasiakos et al., 2015). Fatigue resulting from inadequate recovery is associated with physiological and psychological perturbations that may include endocrine disturbances, inflammation, and a suppressed immune response (Armstrong et al., 2022; Buyse et al., 2019; Cadegiani & Kater, 2017; Carrard et al., 2022; Margonis et al., 2007; Smith, 2000). These perturbations can increase susceptibility to infections, illnesses, and other immune‐related issues (Armstrong et al., 2022; Smith, 2000). In addition, increases in the inflammatory response and oxidative stress can result in significant elevations in muscle degradation, leading to a reduction in muscle protein balance (Meeusen et al., 2013; Nindl et al., 1997).

The use of exogenous anabolic‐androgenic steroids (AAS) may be a potential intervention for combat soldiers participating in sustained military operations and has recently been examined by the United States Military (Pasiakos et al., 2019; Varanoske et al., 2022). However, considering the potential risks associated with AAS administration (Bhasin et al., 2021; Hoffman et al., 2009), greater research is warranted. Recent research has demonstrated that when AAS are provided at physiological levels during 3–4 weeks of simulated military activity such as intense endurance exercise with dietary restriction, lean body mass was preserved but physical performance measures were not maintained (Pasiakos et al., 2019; Varanoske et al., 2022). However, combat more closely resembles anaerobic/strength activity rather than endurance activity (Kraemer & Szivak, 2012). As such, the use of AAS in a model of training that more closely resembles that of a strength/power athlete may be more appropriate to use. Thus, the purpose of this study was to examine the effect of androgen administration on attenuating fatigue when training volume is significantly elevated in mice.

## 2 MATERIALS AND METHODS

### 2.1 Animals

A total of 24 male C57Bl/6J mice (Envigo, Jerusalem, Israel), 3 months of age, were habituated to housing conditions for at least 7 days. For this strain of mice, the equivalent age in a human is approximately 20 years (Dutta & Sengupta, 2016; Flurkey et al., 2007). All mice were housed four per cage in a vivarium with stable temperature (21°C) and a reversed 12‐h light/dark cycle with unlimited access to food (S2018, Envigo, Jerusalem, Israel) and water. The mice were randomly separated into three groups. Two groups of animals (*n* = 8 per group) performed a 3 days per week resistance training program for 3 weeks, while the third group of animals (*n* = 8) remained sedentary and served as the control group (C). The two groups of exercising animals were further randomized into an androgen group (RTA: *n* = 8) and a sham group (RTS: *n* = 8). Following randomization, each group increased training frequency from 3 to 6 days per week to create a volume‐overload exercise stimulus. Mice were handled once daily. Animals were weighed prior to the familiarization phase and at the end of the intervention. This study was performed according to the principles and guidelines of the National Institute of Health Guide for the care and Use of Laboratory Animals. All treatment and testing procedures were approved by the Animal Care Committee of Ariel University (AU – IL – 2310 – 114).

### 2.2 Experimental design

**FIGURE 1:**
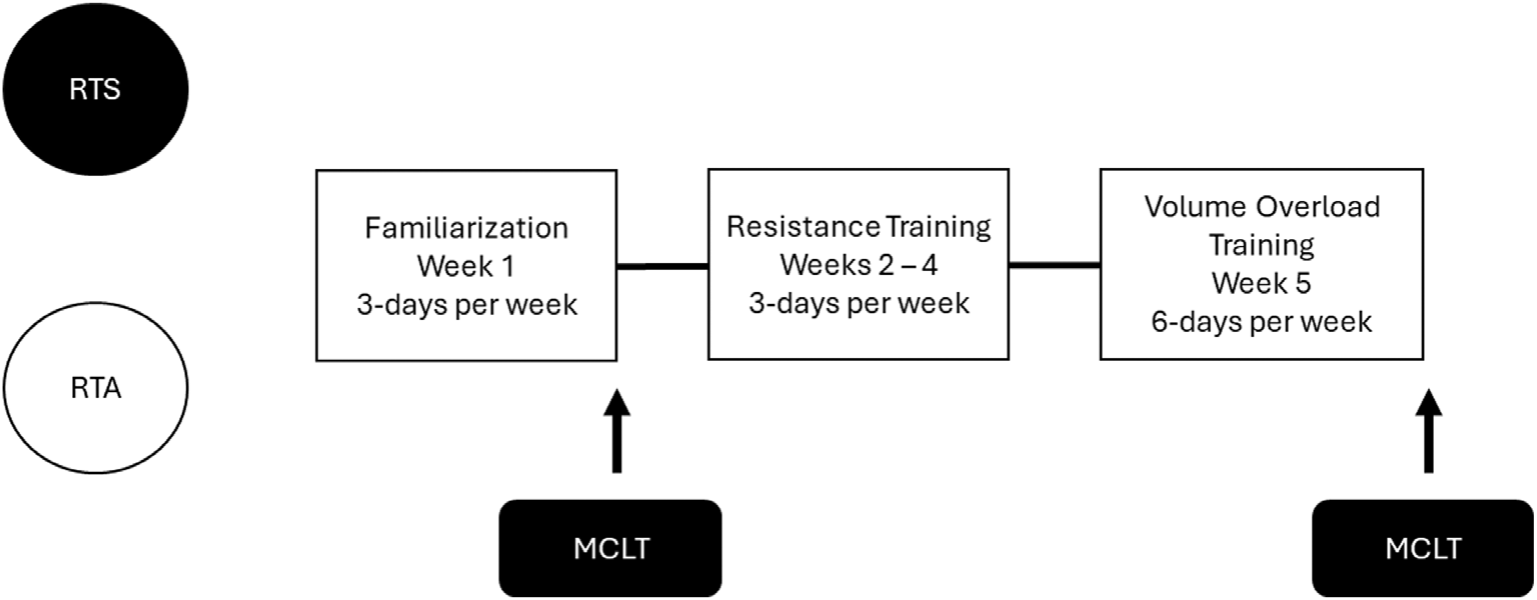
Timeline for resistance training program.

A ladder‐climbing exercise model was employed as the resistance for this study. This model requires the animals to climb a 1‐m ladder at an 85° angle with 1.5 cm between each step (Kim et al., 2015; Krause Neto et al., 2022). The timeline for the resistance training program is depicted in Figure 1. The exercise protocol was initiated without any resistance. This was considered the familiarization phase and was based on a previously published protocol (Cassilhas et al., 2013). During this training phase, the mice climbed the ladder three times, starting from different positions (upper, middle, and base). Between each climb, the mice rested for 60 s. This protocol was performed for five consecutive days. Following the familiarization phase, a maximum load carrying test was conducted to assess the maximum carrying load of a single ladder climb as suggested by Deus et al. (2012). During the maximum load carrying test, weight was attached to a small bottle tied to the mouse's tail. The initial starting weight for the test was 10% of the mouse's body mass, and after every successful climb, the load was increased by an additional 10% of body mass until failure. A 2‐min rest period was provided between each attempt until failure. Failure was recognized as two consecutive unsuccessful attempts to climb the ladder. Maximum carrying load was identified as the highest load carried.

Following the familiarization week, the mice in both the RTS and RTA groups performed the ladder‐based resistance training three times per week, with 48 h between each training session for 3 weeks. Each session required the mice to perform 8 sets of the ladder climb with 2 min of rest between each climb. The protocol required that each mouse perform two sets using a load equal to 50%, 75%, and 85% of their maximum carrying load. A single set with 100% of their maximum carrying load was then performed. If successful, 10% of their body mass was added to their tail, and the mouse performed an additional climb (a total of 8 climbs were conducted per session). If successful, that weight became their new maximum carrying load. If not successful, the same resistance was maintained for the next training session. All training sessions were recorded in a logbook. This resistance training protocol was based upon previous work that demonstrated significant increases in muscle mass, muscle fiber cross‐sectional area, and maximum strength in mice using this protocol (Krause Neto et al., 2022).

The volume overload to create fatigue was employed during the fifth week of training. Both RTS and RTA groups performed double the training sessions from 3 days per week with a 48‐h recovery period to six consecutive days. The exercise protocol for each training session was the same as that used in the previous 3 weeks. During these sessions, if the animals were unable to successfully complete the climb, there was no change in the loading for the following training session. Regardless of their success, all animals attempted 8 sets for each session during the volume overload week. The last successful climb in the last training session was recorded as their final maximum carrying load.

### 2.3 Nandrolone decanoate administration protocol

Forty‐eight hours prior to the initial workout of week five (e.g., volume overload week) mice in the RTA group were injected with nandrolone decanoate (GC63095, GLPBIO, Montclair, CA, USA). Previous research has suggested that a single dose may not be sufficient to increase anabolism, and a second dose may be required (Church et al., 2019). Therefore, to ensure that the mice in RTA increased anabolism during the volume overload training period, a second dose was provided following the last training session. The recommended therapeutic dose of nandrolone decanoate is 0.4 mg·kg^−1^ body mass in humans (Tamaki et al., 2003). Nandrolone decanoate is one of the most widely used AAS because of its efficacy as an anabolic agent, relatively favorable safety profile, and its low affinity for aromatization to estrogen (Hoffman et al., 2009). Most individuals self‐administering AAS use doses that are typically 5–29‐fold greater than a physiological replacement dose (Hoffman et al., 2009). Therefore, we selected a dose approximately 8‐fold higher than the recommended therapeutic dose and then determined the animal equivalent dose (Nair & Jacob, 2016). This was based upon a 6‐fold decrease in resting testosterone concentrations previously reported in soldiers performing high‐intensity, sustained training (Nindl et al., 2007). Our goal was to provide a dose that could be considered a replacement dose during such situational needs. As such, a supraphysiological dose of 38 mg·kg^−1^ body mass was injected intraperitoneally in the mice of the RTA group. Mice from RTS and C were injected with sesame oil (#241000010, Acros Organics, Belgium) at the same frequency. The animals were under daily surveillance to detect any behavioral changes.

### 2.4 Determination of fatigue based on performance measures

There is no clear consensus regarding the determination of fatigue. Although performance measures are suggested to represent the most direct and specific outcome that can be used (Kellmann et al., 2018), it is generally acknowledged that fatigue and recovery is a multifocal event that lacks a simple definition (Kellmann et al., 2018). For instance, a decrease in performance of any magnitude is undesirable and considered to be the gold standard (Bell et al., 2021), but does a performance decrease truly represent a real performance decrement or is it part of the normal day to day variability of performance? As such, it was decided to use the “smallest real difference” (SRD) to provide a numerical value that delineates what should be the clinically smallest meaningful change at the level from what is known as the error of measurement (Dvir, 2015). The SRD for an individual response is determined by the standard error of measurement of the change score (∆ score) multiplied by 2.77 (Dvir, 2015). Animals whose ∆ score (volume overload week – week three of the training program) in both the MCL and average daily training volume from the volume overload week compared to week three of training that exceeded the SRD were determined to be fatigued.

### 2.5 Tissue preparation

Twenty‐four hours following the last training session, the mice were deeply anesthetized using 400 μL of 20 mg/mL^−1^ Pentobarbital sodium (CTS Chemical Industries, Israel), injected IP. The chest was cut open and blood was obtained from the left ventricle for further assessments. The animals were then perfused transcardially with phosphate‐buffered saline (PBS). The hind limbs of every animal were cut at the hip joint and the skin was removed. The soleus and plantaris muscles were extracted from both hind limbs. Each muscle was put into a 1.5 mL Eppendorf tube, snap frozen in liquid nitrogen, and stored at −80°C until further analysis.

### 2.6 Western blot analysis

The plantaris and soleus muscles from each animal were homogenized in RIPA lysis buffer (Merck, Germany) and protease inhibitor cocktail (#539134, Calbiochem, USA) at a 1:100 dilution factor. Protein concentration was evaluated using a BCA kit (Merck, Germany) according to the manufacturer's instructions. Samples were denatured by adding β‐mercaptoethanol (Merck, Germany) mixed with sample buffer (Bio‐Rad, USA) at a ratio of 1:10, and boiled at 70°C for 10 min. The denatured protein content of the homogenates was separated using sodium dodecyl sulfate‐polyacrylamide gel electrophoresis (SDS‐PAGE). In addition, a prestained protein ladder (Abcam, MA USA) was loaded onto the gel to confirm the size of the proteins of interest. Separated proteins were transferred from the gel to a nitrocellulose membrane using a transfer pack (Bio‐Rad, USA) and a turbo transfer device (Bio‐Rad, USA). After completion of the transfer, the membrane was washed with Ponceau S solution (Abcam, Cambridge, England) to verify the completeness of the transfer. The membrane was then incubated in a blocking solution with 5% BSA diluted in PBS for 1 h at room temperature. Following blocking incubation, the membrane was incubated overnight at 4°C in a cocktail of primary antibodies containing PBS with 5% BSA, rabbit anti‐androgen receptor (AR, Bioss, bs‐0118R) diluted 1:1000, rabbit anti‐tumor necrosis factor‐α (TNFα, Bioss, bs‐0078R) diluted 1:500, mouse anti‐ glucocorticoid receptor (GR, Santa Cruz, sc‐393232) diluted 1:1000, mouse anti‐Glutathione peroxidase (GPX, Santa Cruz, sc‐166,570) diluted 1:1000, and mouse anti‐ α tubulin diluted 1:1000 (Santa Cruz, sc‐5286). On the second day, the membrane was washed three times for 5 min with tris buffer saline‐tween (Bio‐Lab, Jerusalem, Israel) before being incubated for 1 h at room temperature in a cocktail of fluorescent secondary antibodies containing PBS with 5% BSA, anti‐mouse Alexa Fluor® 680 (Jackson ImmunoResearch, 711‐655‐152) diluted 1:10000, and anti‐rabbit Alexa Fluor® 790 (Jackson ImmunoResearch, 715‐625‐150) diluted 1:10000. The membrane was rewashed as described and scanned for protein visualization and analysis using the Odyssey CLx system (LI‐COR, Nebraska USA) resolution: 169 μm; intensity: auto mode; according to the manufacturer's instructions. The fluorescence intensity of proteins of interest bands was determined using the Odyssey Infrared Imaging System software (Image StudioV5.2, Li‐Cor Biosciences, Lincoln, NE, USA). Protein expression was calculated as a percentage of the α‐tubulin fluorescence intensity.

### 2.7 Muscle protein synthesis measures

Twenty‐four hours prior to the animals being euthanized, they were injected IP with 36.5 μL of deuterated water (^2^H_2_O; 70% enriched; Cambridge Isotope Laboratories, Andover, MA, #7789‐20‐0) per gram of body weight (Cross et al., 2020).

#### 2.7.1 Analytical methods

To determine muscle protein synthesis, approximately 3.57 mg for soleus and 8.66 mg for plantaris muscle wet weight was used for analysis. To each sample, 800 μL of 7.5% Sulfosalicylic acid was added, followed by grinding the tissue using a battery‐operated pestle device. Once homogenized, the tissue was centrifuged at 14800*g* for 10 min at 4°C. The supernatant was collected and set aside. This step was repeated once more. The supernatant, known as the intracellular fraction, was then stored at −80°C for later analysis.

Once the intracellular fraction was aliquoted off, 800 μL of 7.5% Sulfosalicylic acid was added, and the sample was then vortexed and centrifuged again. This time the supernatant was discarded. This was repeated twice more. Ethanol was then added to the sample. The sample was again vortexed and centrifuged, and again the supernatant was discarded. This step was repeated once. For the last step, 1 mL ethyl ether was added and the sample was vortexed, centrifuged, and then the supernatant was discarded. The sample was then dried in an oven overnight at 50°C. Once dry, the sample was weighed (approximately 0.84 mg for soleus and 2.17 mg for plantaris muscle dry weight) and transferred to a 13 × 100mL tube. 3 mL of 6 N HCl was added, and the tube was placed on a dry bath for 24 h at 120°C.

For isotope isolation, each sample was passed through a cation exchange column (Phenomenex, Strate‐X‐C, 60 mg, Torrance, CA). In brief, each tube was activated with 1 mL methanol, washed with water, conditioned for a specific sample, washed with water, and collected using a 95% methanol to 5% ammonium hydroxide solution. The sample was then placed in a speedvac and dried overnight. Once dry, the sample was derivatized with an FMOC solution and analyzed on a QTRAP 5500 triple quad system LC–MS (ABSCIEX, Redwood, CA) (Cross et al., 2020).

#### 2.7.2 Calculations

Based on previous work (Cross et al., 2020), we chose to analyze the true precursor of the incorporation into protein alanine rather than an indirect measurement. We calculated the labeled to unlabeled ratio (tracer‐tracee ratio) of alanine of each muscle and corrected the value with the natural abundance of the tracer. With the corrected tracer‐tracee ratio, we calculated the protein fractional synthetic rate (FSR) with the following equation:

$$\mathrm{FSR}=\frac{-\ln\left(1-f\right)}{t}$$

where *f* is calculated as the protein‐bound alanine tracer‐tracee ratio divided by the intracellular fraction alanine tracer‐tracee ratio, and *t* is the amount of time between the bolus administration of deuterated water and the collection of tissue, that is, 1 day. FSR is expressed as fraction new/day (F/D).

### 2.8 Blood assessments

Serum testosterone was measured using a testosterone ELISA Kit (Arbor Assays, K032‐H1), according to the manufacturer's instructions. Briefly, serum was separated from the blood obtained from mice. Diethyl ether, at a ratio of 5:1 to serum, was added to the samples, and the mixture was dried out using an Eppendorf concentrator 5301 (Eppendorf, Germany) at 4200 rpm. The dried samples were reconstituted with assay buffer and loaded in duplicates onto 96‐well plates. A microplate reader (Infinite m plex, Tecan Inc., Switzerland) was utilized to determine the absorbance at 450 nm. The mean intra‐assay variability was <10%.

### 2.9 Statistical analyses

To compare the effect of nandrolone decanoate administration on changes in maximal carrying load and average volume‐load, a delta (∆) was determined (volume overload week performance – week 3 performance). A student's unpaired *t*‐test examined the differences in ∆ scores between groups. To examine the occurrence of fatigue, a *χ* ^2^ analysis was used to compare the individual animal responses that exceeded the SRD between RTA and RTS. For all biological measures, a one‐way analysis of variance was used to compare all molecular results between RTA, RTS, and CTL. In the event of a significant F ratio, a Bonferroni post hoc analysis was used for pairwise comparisons. Due to the novel nature of the animal model of fatigue used in this study, an a priori sample size calculation was not performed. All statistical analyses were analyzed using SPSS v29 software (SPSS Inc., Chicago, IL, USA), and an α level of *p* ≤ 0.05 was used to determine statistical significance. Performance and body mass data are reported as mean ± SD, while all biological data are reported as mean ± SEM.

## 3 RESULTS

**FIGURE 2:**
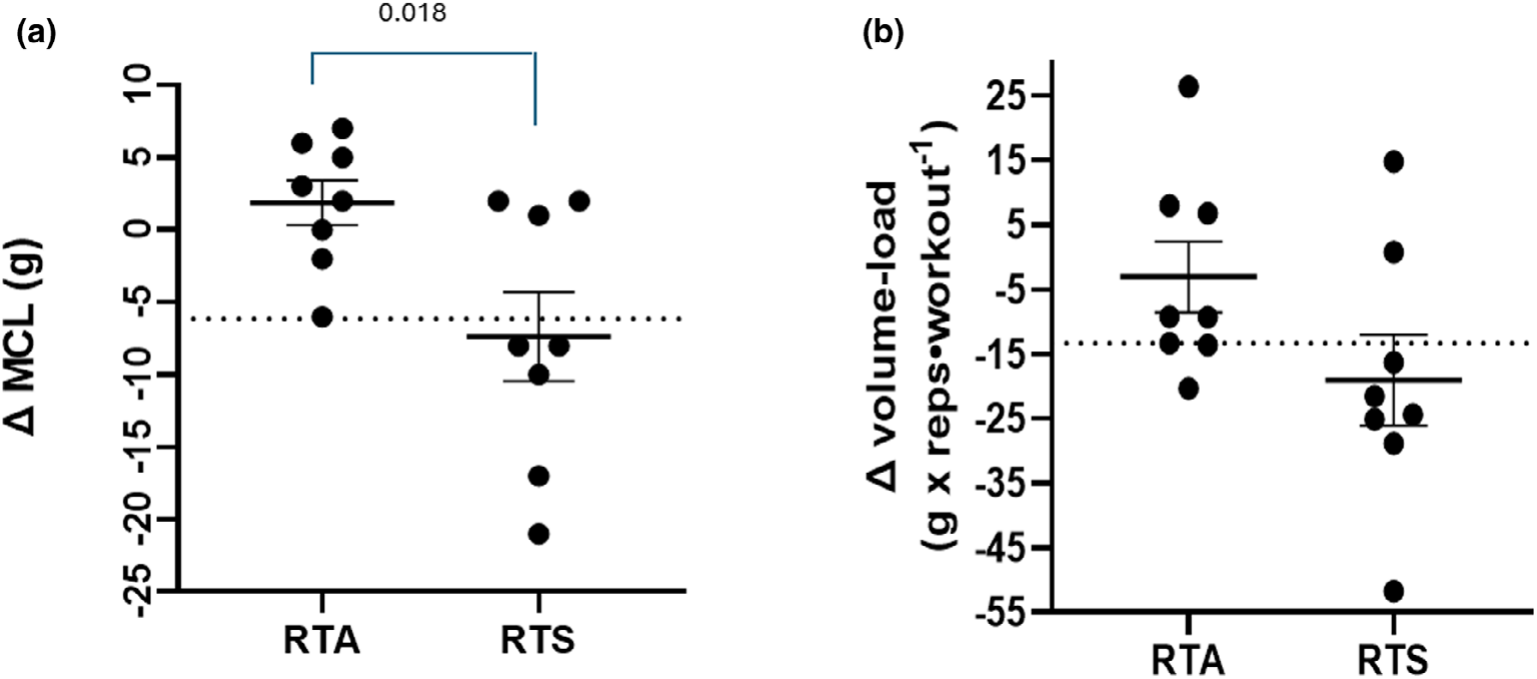
Individual ∆ (Volume Overload week – Week 3) Performance Results for MCL (a) and volume‐load (b). Dotted line for each graph represents the smallest real difference (SRD) for each measure. Animals whose performance was below that line were considered fatigued. The SRD for MCL was 6.15 g and for volume‐load was 13.3 g × reps· workout^−1^. ∆ MCL, change in maximal carrying load; RTS, resistance training sham; RTA, resistance training androgen administration.

Total weekly training volume was significantly elevated (*p* < 0.001) from week 3 (498 ± 82 g) to the volume overload week (929 ± 138 g), but no differences (*p* = 0.31) were noted between total volume performed between RTA and RTS during the volume overload week. Examination of the individual ∆ scores for MCL and volume‐load in both RTA and RTS can be observed in Figure 2a,b, respectively. The average ∆ score for maximal carrying load was significantly greater for RTA (1.9 ± 4.4 g) compared to RTS (−7.4 ± 8.7 g). Differences between RTA (−3.1 ± 15.5 g × reps‧workout^−1^) and RTS (−19.0 ± 19.9 g × reps‧workout^−1^) in the average ∆ score for volume‐load did not result in a significant difference (*p* = 0.095). The determination of the individual animals who were fatigued was based on negative changes in the ∆ performance (volume overload week – week 3) that exceeded the SRD for maximum carrying load and average daily volume‐load. The SRD for maximum carrying load was 6.149 g (SEM 2.22 × 2.77). Five mice in RTS had changes in maximum carrying load performance that exceeded the SRD for maximum carrying load, while one mouse in RTA had a change that exceeded the SRD. *χ* ^2^ analysis revealed that the occurrence of fatigue in strength performance between the groups was significantly different (*p* = 0.033). Examination of the changes in the average volume‐load per workout for the volume overload week indicated that six of the eight mice in RTS had a decrease in the average volume‐load per workout that was greater than the SRD (13.3 g × reps‧workout^−1^) compared to three mice in the RTA. χ^2^ analysis revealed the frequency of this occurrence was not significantly different between the groups (*p* = 0.125). Four mice experienced decreases in both maximum carrying load and average volume‐load that exceeded the SRD while none of the mice in RTA had decreases that exceeded the SRD in both performance measures. *χ* ^2^ analysis revealed that this difference between groups was significantly different (*p* = 0.009).

Examination of the change in body mass from familiarization to the end of the volume‐overload week of training revealed a 0.53 ± 0.77 g increase in RTS and a 1.01 ± 1.82 g and 1.05 ± 1.82 g increase in RTA and CTL, respectively. Comparisons of changes in body mass revealed no significant differences (*F* = 0.460, *p* = 0.505) between the groups. Examination of the individual changes in body mass following the end of the volume‐overload week revealed that the body mass of three mice within RTS, two mice within RTA, and only one mouse in CTL was lower than their pre‐study body mass. *Χ* ^2^ analysis examining the number of animals losing versus gaining mass revealed no significant differences (*p* = 0.513).

**FIGURE 3:**
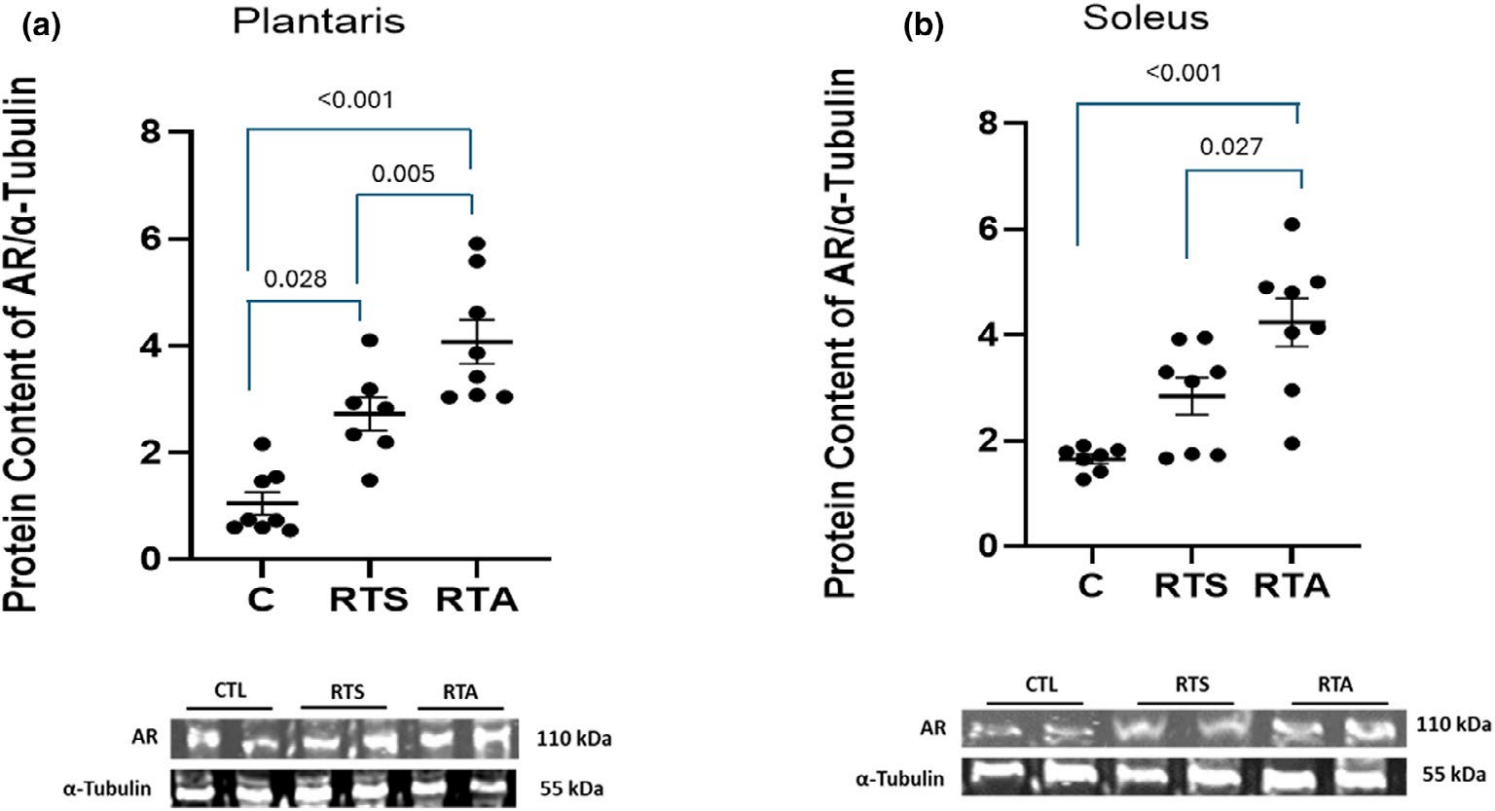
Androgen receptor (AR) expression (a) plantaris and (b) soleus muscles. C, control group; RTS, resistance training sham; RTA, resistance training androgen administration. Representative Western blot images are shown below the quantified data. Original images can be found in Appendix S1. Individual animal responses and group mean ± SEM are reported.

**FIGURE 4:**
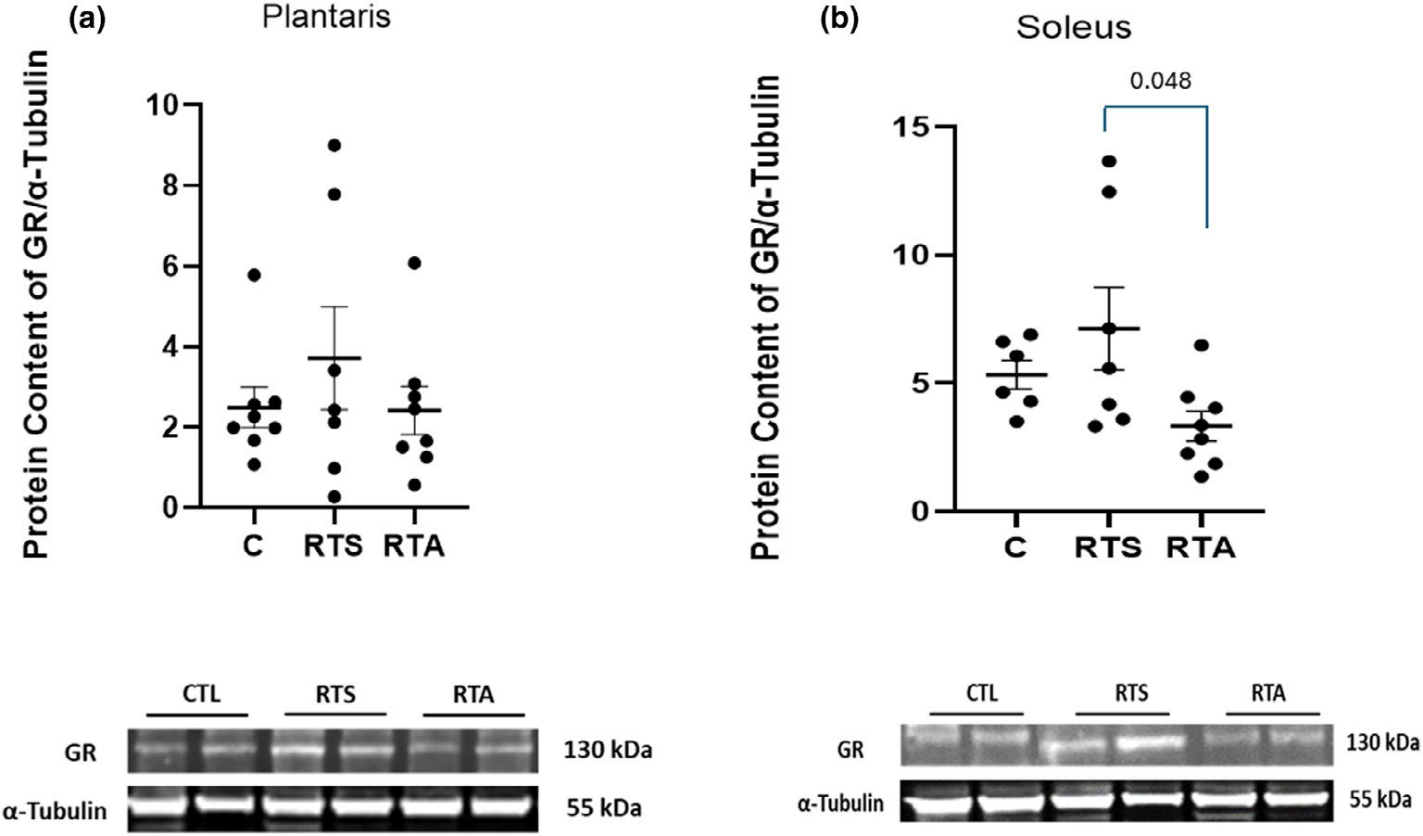
Glucocorticoid receptor (GR) expression (a) plantaris and (b) soleus muscles. C, control group; RTS, resistance training sham; RTA, resistance training androgen administration. Representative Western blot images are shown below the quantified data. Original images can be found in Appendix S1. Individual animal responses and group mean ± SEM are reported.

Expression of AR and GR in the plantaris and soleus muscles are depicted in Figures 3 and 4, respectively. One‐way ANOVA revealed a significant difference in AR expression of both the plantaris (*F* = 22.5, *p* = < 0.001) and soleus (*F* = 13.41, *p* < 0.001) muscles. AR expression in the plantaris was significantly greater for RTA compared to both C and RTS (see Figure 3a). In addition, AR expression for RTS was significantly greater than C. In the soleus, AR expression was significantly greater for RTA than C and RTS (see Figure 3b). No differences were noted in GR expression in the plantaris (*F* = 0.758, *p* = 0.482) (see Figure 4a), but a significant difference was noted in GR expression in the soleus (*F* = 3.545, *p* = 0.050). GR expression was significantly greater in RTS than RTA (see Figure 4b). No other significant differences were observed.

**FIGURE 5:**
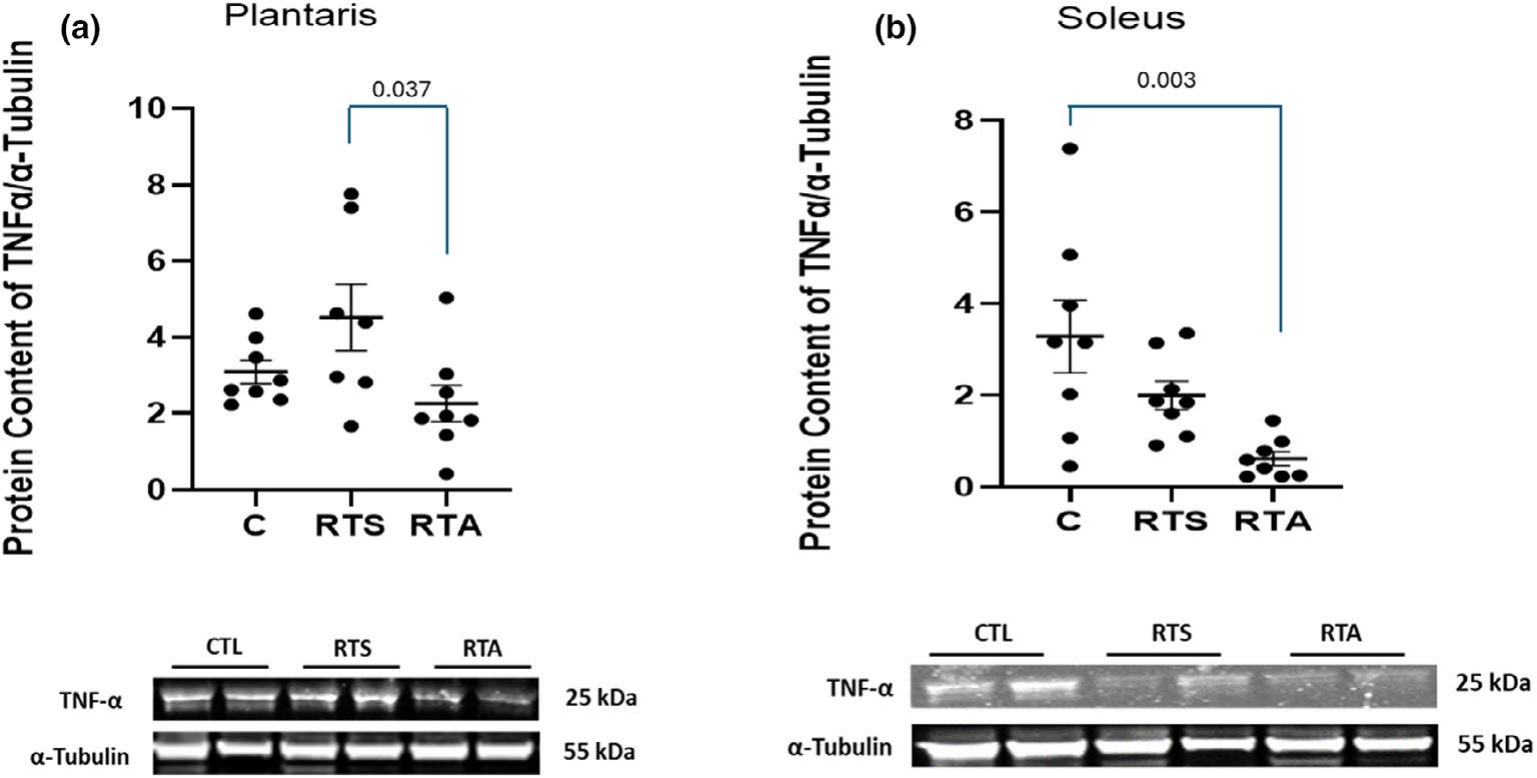
Tumor necrosis factor‐α (TNFα) expression (a) plantaris and (b) soleus muscles. C, control group; RTS, resistance training sham; RTA, resistance training androgen administration. Representative Western blot images are shown below the quantified data. Original images can be found in Appendix S1. Individual animal responses and group mean ± SEM are reported.

**FIGURE 6:**
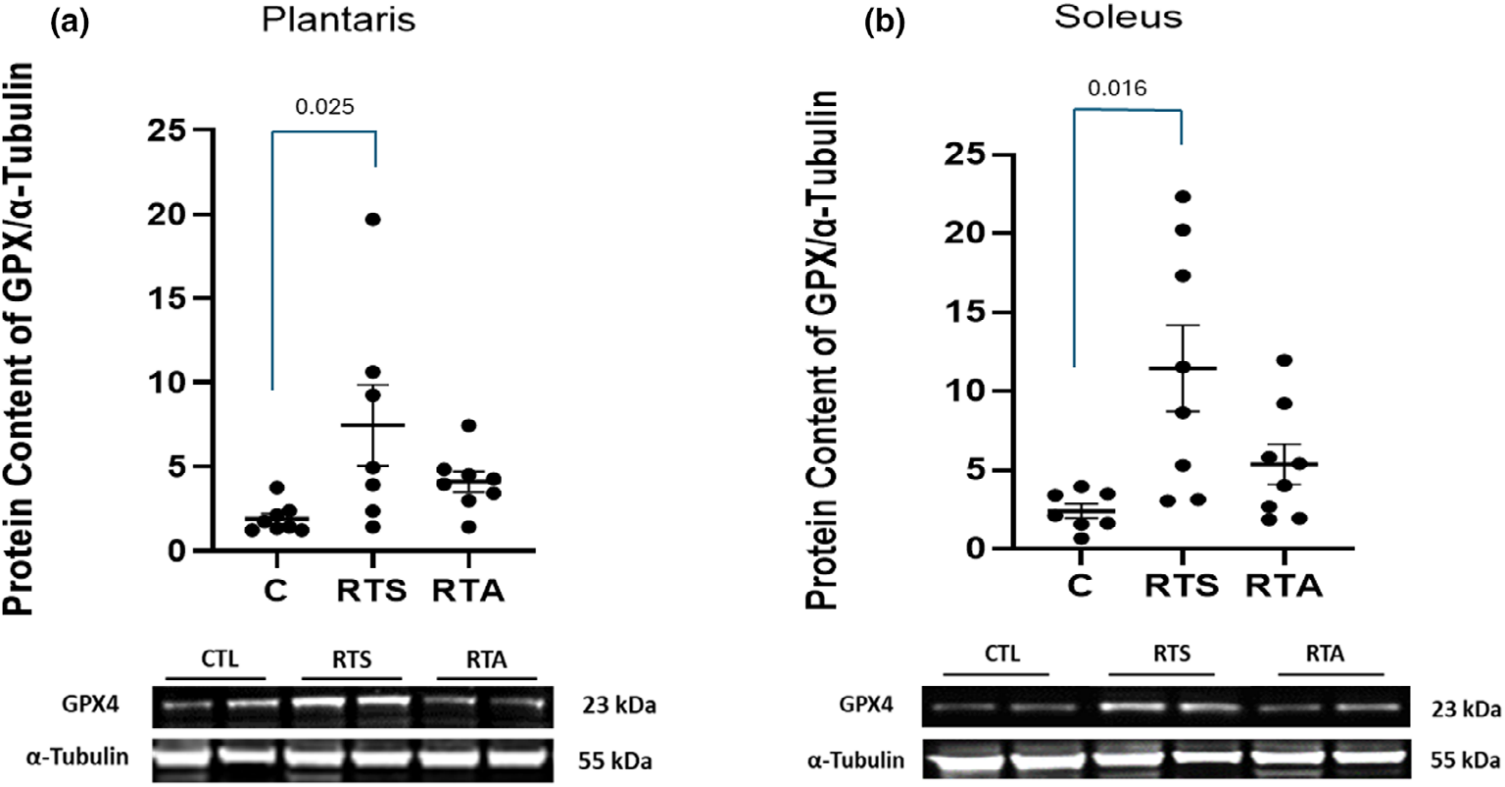
Glutathione peroxidase (GPX) expression in (a) plantaris and (b) soleus muscles. C, control group; RTS, resistance training sham; RTA, resistance training androgen administration. Representative Western blot images are shown below the quantified data. Original images can be found in Appendix S1. Individual animal responses and group mean ± SEM are reported.

Expressions of TNFα and GPX in the plantaris and soleus muscles are depicted in Figures 5 and 6, respectively. One‐way ANOVA revealed significant differences in TNFα expression in both the plantaris (*F* = 3.819, *p* = 0.039) and soleus (*F* = 7.155, *p* = 0.004) muscles (see Figure 4a,b, respectively). Post hoc analysis revealed that TNFα expression in the plantaris was significantly lower for RTA compared to RTS, while TNFα expression in the soleus was significantly lower for RTA compared to C. No other differences for TNFα expression within the plantaris or soleus were noted. A significant difference in GPX expression was observed in both the plantaris (*F* = 4.298, *p* = 0.028) and soleus (*F* = 6.275, *p* = 0.008) muscles (see Figure 6a,b, respectively). GPX expression was significantly greater for RTS compared to C in the plantaris and soleus (*p* = 0.009) muscles.

**FIGURE 7:**
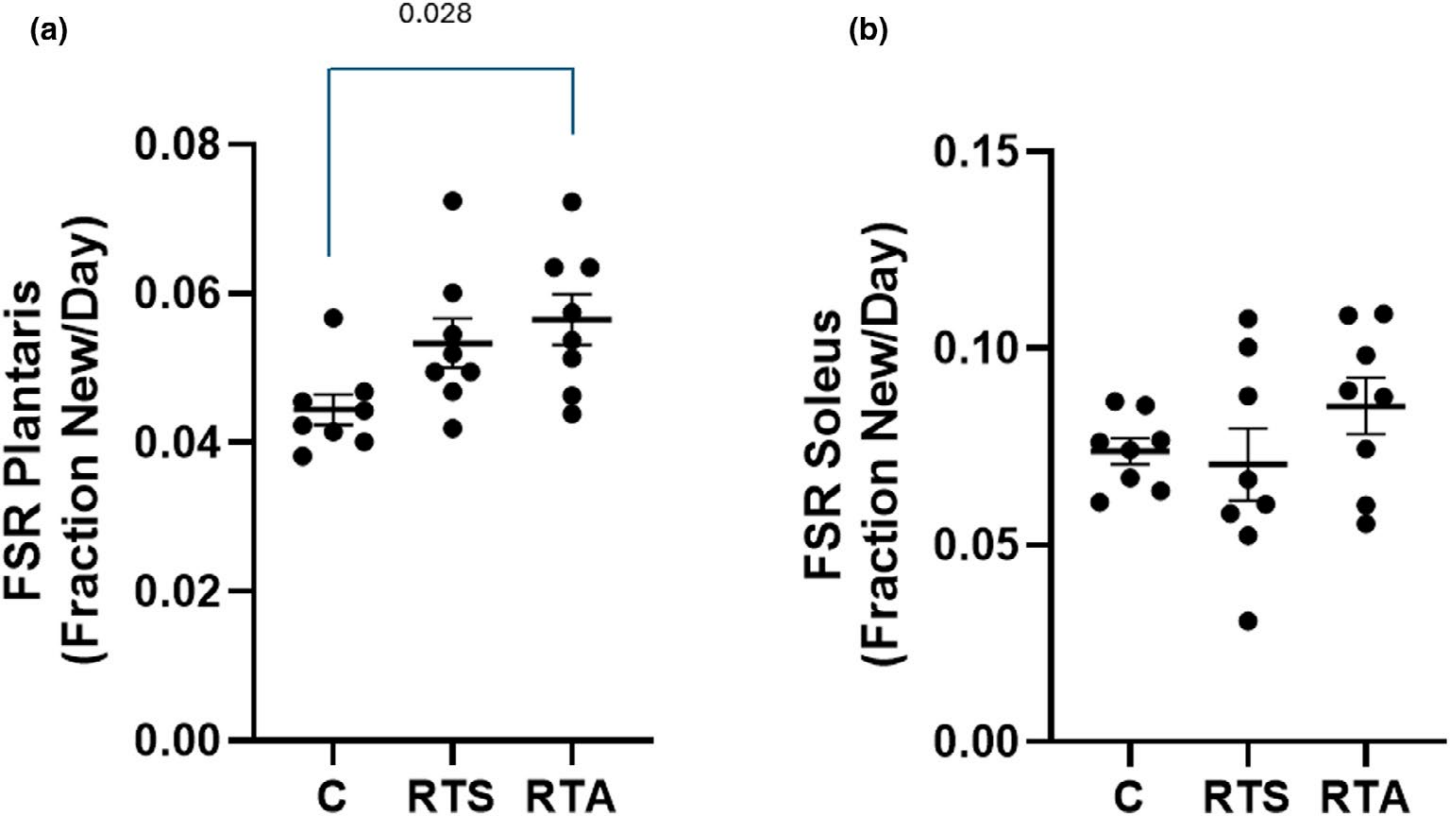
Fractional synthesis rate in (a) plantaris and (b) soleus muscles. C, control group; RTS, resistance training sham; RTA, resistance training androgen administration. Individual animal responses and group mean ± SEM are reported.

Protein synthesis rates of the plantaris and soleus muscle are depicted in Figure 7a,b, respectively. A significant difference was noted in the plantaris muscle (*F* = 4.408, *p* = 0.025) but not in the soleus muscle (*F* = 1.224, *p* = 0.314). Post hoc analysis revealed that the FSR for RTA was significantly greater than C in the plantaris muscle. No other significant differences were noted.

## 4 DISCUSSION

This study used a novel animal model of resistance training to create the fatigue stimulus. To simulate sustained, high‐intensity activity that would result in exertional fatigue, the training volume was doubled by increasing the frequency of training from 3 days per week to 6 days during the volume overload week of training. This resulted in a significant increase in training volume, as defined by the total number of repetitions (e.g., climbs) performed × load carried. An animal was deemed to be fatigued if the ∆ score of both the maximal carrying load and average volume load was decreased past the SRD. Nandrolone decanoate administration prior to the onset and at the end of the volume overload week resulted in significant increases in circulating androgen concentrations and an increase in androgen receptor upregulation, which appeared to provide an ergogenic benefit for RTA. Only one of the mice in RTA experienced a decline in maximum carrying load that exceeded the SRD, while five of the mice in RTS experienced a decline in maximum carrying load that exceeded the SRD. Interestingly, the mouse in RTA that had a decrease in maximum carrying load that reached the SRD had the lowest circulating testosterone concentrations of the group. Further, four mice in RTS had decreases in both maximum carrying load and average training volume that exceeded the SRD, while none of the animals in RTA experienced changes in both measures that exceeded the SRD. Thus, the nandrolone decanoate intervention did appear to attenuate the fatigue response. The ergogenic benefits experienced by RTA were also supported by the biological measures reported. Mice provided with nandrolone decanoate experienced significant reductions in TNF‐α in both the plantaris and soleus muscle. TNF‐α is a marker of tissue inflammation. In addition, RTS experienced a significant elevation in oxidative stress compared to C in the plantaris, but no differences were noted between RTA and C, suggesting a lower oxidative stress response as a result of nandrolone decanoate intervention. Further, GR expression was significantly lower in the soleus muscle of RTA compared to both RTS and C, further suggesting a lower catabolic response to the volume‐overload training paradigm.

Increases in fatigue as the result of an exercise stimulus has been reported to cause significant increases in the inflammatory response (Smith, 2000). The TNF‐α response within the plantaris muscle observed in RTS is consistent with those reports. There is limited research that has examined the effect of exogenous androgen administration on attenuating the inflammatory response to exercise. Roberts and colleagues (Roberts et al., 2014) examined five different weekly doses of testosterone enanthate, ranging from physiological to supraphysiological levels (25–600 mg) for 20 weeks and reported no changes in C‐reactive protein, a systemic inflammatory marker. The participants of the study were young but were not provided any physical stress during the study period. This study appears to be the first examination on the effect of exogenous androgen use on the inflammatory response to an exertional fatiguing stimulus. The significant differences observed between RTS and RTA in TNF‐α expression in the plantaris during the volume overload week suggest that short‐term exogenous nandrolone decanoate administration may provide an anti‐inflammatory effect during stressful training. Variability in the response between the plantaris and soleus is likely a function of the differences in fiber type between the muscles. The plantaris is comprised primarily of fast‐twitch fibers, whereas the soleus is primarily composed of slow‐twitch fibers (Kuhnen et al., 2022). Muscle fiber recruitment during resistance training is based upon the size principle, with high‐threshold fibers primarily recruited during high‐intensity training (Campos et al., 2002). Although speculative, the greater inflammatory response observed in the plantaris muscle compared to the soleus likely reflects this recruitment pattern. Further, these results are also consistent with other investigations that indicated a differential response to androgen intervention between oxidative and glycolytic muscle groups (Asfour et al., 2021).

In addition to the inflammatory response, we were also interested in examining the oxidative stress response to the training stimulus. Increases in oxidative stress have also been associated with exertional fatigue (Joro et al., 2020; Margonis et al., 2007). The significant elevation in GPX observed in RTS in the plantaris muscle and the trend towards a difference in the soleus muscle are consistent with the oxidative stress response associated with exertional fatigue. GPX is an enzyme responsible for reducing H_2_O_2_ or organic hydroperoxides to water and alcohol, respectively (Margonis et al., 2007). Its elevation reflects greater oxidative stress within the tissue as its role is to act as a protectant against potential tissue damage (Margonis et al., 2007). The results of this study are also consistent with the investigation of (Saborido et al., 2011) who demonstrated a significant reduction in oxidative stress in an acute exercise stress in rats treated with androgens. They suggested that androgen intervention may reduce reactive oxygen species generated by mitochondria during muscle contraction, leading to reduced tissue damage. This is supported by Nikolic and colleagues (Nikolic et al., 2016) who reported that exercise training in rats will initially result in a significant elevation in oxidative stress, which is slightly elevated with the administration of a supraphysiological dose of nandrolone decanoate. However, within 4 weeks of the exercise and androgen protocol, the oxidative stress response was significantly lowered. It was suggested that the antioxidative effects of exercise provided some compensatory effect over the duration of the study. Others have demonstrated that chronic AAS use in human subjects results in a greater oxidative stress response at rest and to a resistance exercise training session (Arazi et al., 2017). It is possible that prolonged AAS use (>1 year) may result in a different oxidative stress response than an acute administration to increase resiliency. Further research is still needed to provide a greater understanding of this complex molecular interaction.

Nandrolone decanoate intervention resulted in a significant up‐regulation of AR expression in both the soleus and plantaris muscles. Resistance training had a significant impact on the upregulation of the AR in the plantaris muscle only. This is supported by previous research indicating that resistance training is a potent stimulus for upregulation of the AR (Ahtiainen et al., 2011). Although speculative, the high‐intensity training program likely preferentially recruited the plantaris muscle group due to its high percentage of fast‐twitch fiber composition (Kuhnen et al., 2022). The supraphysiological dose of nandrolone decanoate appeared to further stimulate a more potent upregulation of AR than training alone and appeared to stimulate an upregulation of AR in the soleus muscle as well. To the best of our knowledge, this appears to be the first study to examine the combined effect of resistance exercise and nandrolone decanoate intervention using a supraphysiological dose. Results show that this combination provides a potent stimulus for upregulating the AR that is significantly greater than training alone.

The use of exogenous AAS ingestion has previously been suggested to antagonize the glucocorticoid effect during exercise (Hickson et al., 1990; Kuhnen et al., 2022). Exertional fatigue has been reported to increase the glucocorticoid response, reflecting the stress associated with excessive training (da Rocha et al., 2017). However, this does not appear to be consistent among all studies. Nicoll and colleagues reported no change in the GR following a nonfunctional overreaching protocol in resistance‐trained men (Nicoll et al., 2019). However, they did report significant elevations in serum cortisol concentrations at the end of the intense training period. It was suggested that these results reflect potentially different mechanisms stimulating the hormonal and tissue response to an exertional exercise stress. Our results are consistent with the latter findings in that no changes in GR were noticed in either RTS or RTA compared to C in the plantaris muscle. However, a significant downregulation was noted in GR for RTA compared to both RTS and C in the soleus muscle. This potentially provides partial support to previous investigations suggesting that AAS intervention can antagonize the GR response within tissue (Hickson et al., 1990; Hoffman et al., 2009). It is not clear why downregulation of the GR would be seen in the soleus muscle and not the plantaris muscle. Speculatively, it is possibly related to a preferential recruitment of the plantaris muscle compared to the soleus muscle during the training stress resulting in a great upregulation of the GR in the plantaris. Further research will need to examine this more thoroughly.

The FSR of both the plantaris and soleus muscles was determined to provide a measure of the anabolic/catabolic effect of the training program and the potential effect that a supraphysiological dose of nandrolone decanoate had on FSR during stressful training. A recent study from this research team demonstrated that 6 weeks of nandrolone decanoate administration in mice resulted in significant elevations in the FSR in the gastrocnemius muscle but not the soleus (Church et al., 2024). In that study, the animals did not perform any exercise program. Previous research has indicated that resistance training is a potent stimulus for increasing protein synthesis (Phillips et al., 1999). Significant elevations in FSR observed in both RTA and RTS suggest that the resistance training program, and not nandrolone decanoate administration, was the overriding stimulus responsible for the elevation in FSR in the plantaris muscle. There appears to have been only a limited number of studies that have previously examined the combination of resistance exercise and AAS administration on muscle FSR. Previously, the addition of testosterone isocaproate administration to resistance exercise was reported to result in a significantly greater increase in protein synthesis rate compared to resistance training only (Gharahdaghi et al., 2019); however, that study was longer in duration (i.e., 6 weeks) than the present study. Interestingly, significant differences in protein synthesis rates were observed only after 3 weeks of testosterone isocaproate use and resistance exercise, indicating that the combination of AAS administration and resistance exercise takes a few weeks to result in a significant response. Considering the purpose of this study was to examine the efficacy of nandrolone decanoate in limiting performance during a volume overload stress, the results regarding differences in FSR are not surprising.

No adverse effects were noted in any of the mice administered nandrolone decanoate. A recent study from our lab showed that 7 weeks of resistance exercise with nandrolone decanoate administration using the same dosing pattern as used in the present study had no adverse effects on cardiac tissue (Atias et al., 2025). However, the examination of all potential side effects associated with AAS administration, and nandrolone decanoate specifically, were not assessed in this study. As such, it is important to acknowledge that several other investigations have reported adverse effects associated with supraphysiological nandrolone decanoate administration (Alves et al., 2024; Ferreira et al., 2025; Shirpoor & Naderi, 2024; Zelleroth et al., 2024), suggesting that further examination regarding the safety profile of nandrolone decanoate dose and duration of use is recommended.

In conclusion, the evidence provided by this study suggests that nandrolone decanoate intervention during a period of volume overload may provide significant ergogenic benefits. Specifically, nandrolone decanoate was able to sustain performance during a period of high volume, high intensity training (analogous to the physiological stress during sustained military operations). Inherent in this process was a mitigation of muscle inflammatory and oxidative stress markers, as well as glucocorticoid expression. Although androgen use is strictly forbidden by all sports governing bodies, the potential use of androgens in the tactical athlete (i.e., soldier), especially during highly intense, sustained combat operations may be a potential consideration for combat medical teams. Considering that this was an animal study demonstrating “proof‐of‐concept”, additional research is warranted.

## AUTHOR CONTRIBUTIONS

TBZ performed experiments, drafted manuscript, and analyzed data. DC analyzed data, edited and revised manuscript. CL, IW, AS, AL, and DS performed experiments. AF performed experiments and analyzed data. AAF analyzed data, edited and revised manuscript. JRH conceived and designed research, analyzed data, edited and revised manuscript, and approved final version of manuscript.

## FUNDING INFORMATION

This work was supported by an internal grant from Ariel University (RA2300000434) and from the Israel Science Foundation (ISF grant no 1984/21).

## ETHICS STATEMENT

This study was performed according to the principles and guidelines of the National Institute of Health Guide for the care and Use of Laboratory Animals. All treatment and testing procedures were approved by the Animal Care Committee of Ariel University (AU – IL – 2310 – 114).

## Supporting information


**Appendix S1.** Western blot plots for all analyses assessed in the plantaris and soleus muscles.

## Data Availability

Data will be made available on request.
