# Supplementary figures and images for: The effect of nandrolone decanoate administration on fatigue during a volume‐overload stress in male mice
Source: Physiol Rep. 2025 May 8;13(9):e70334. doi: 10.14814/phy2.70334 (PMC12059473; doi:10.14814/phy2.70334)

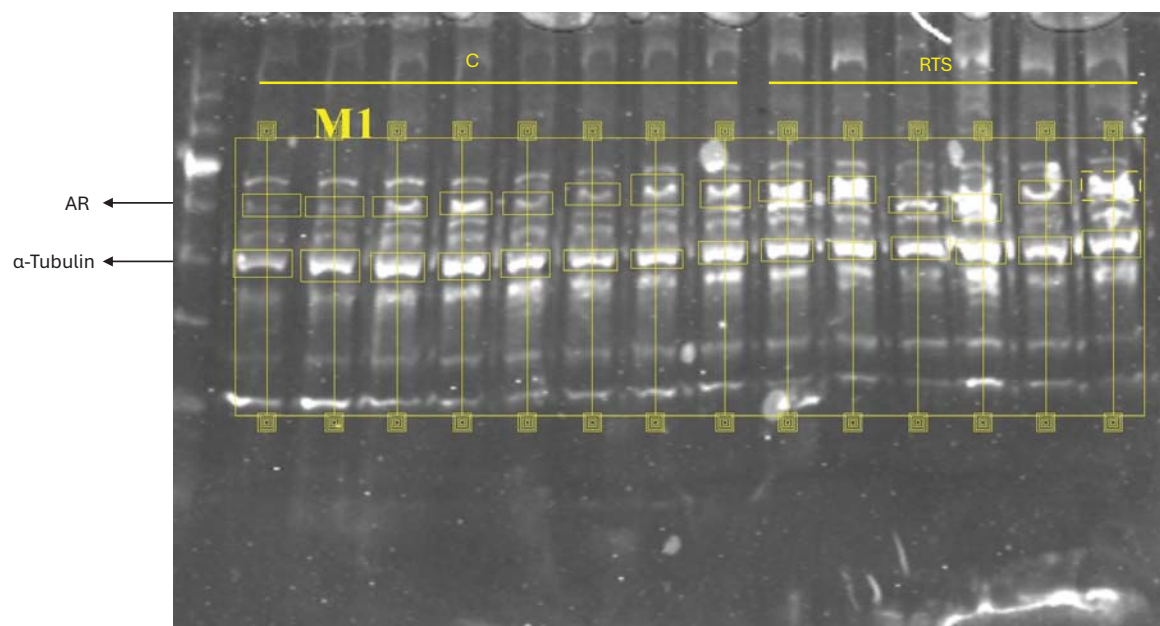

Supplement: Supplementary file 1 — Appendix S1. Western blot plots for all analyses assessed in the plantaris and soleus muscles. [file PHY2-13-e70334-s001.zip › PHYSREP-2025-02-130-T-f08-z-.pdf]

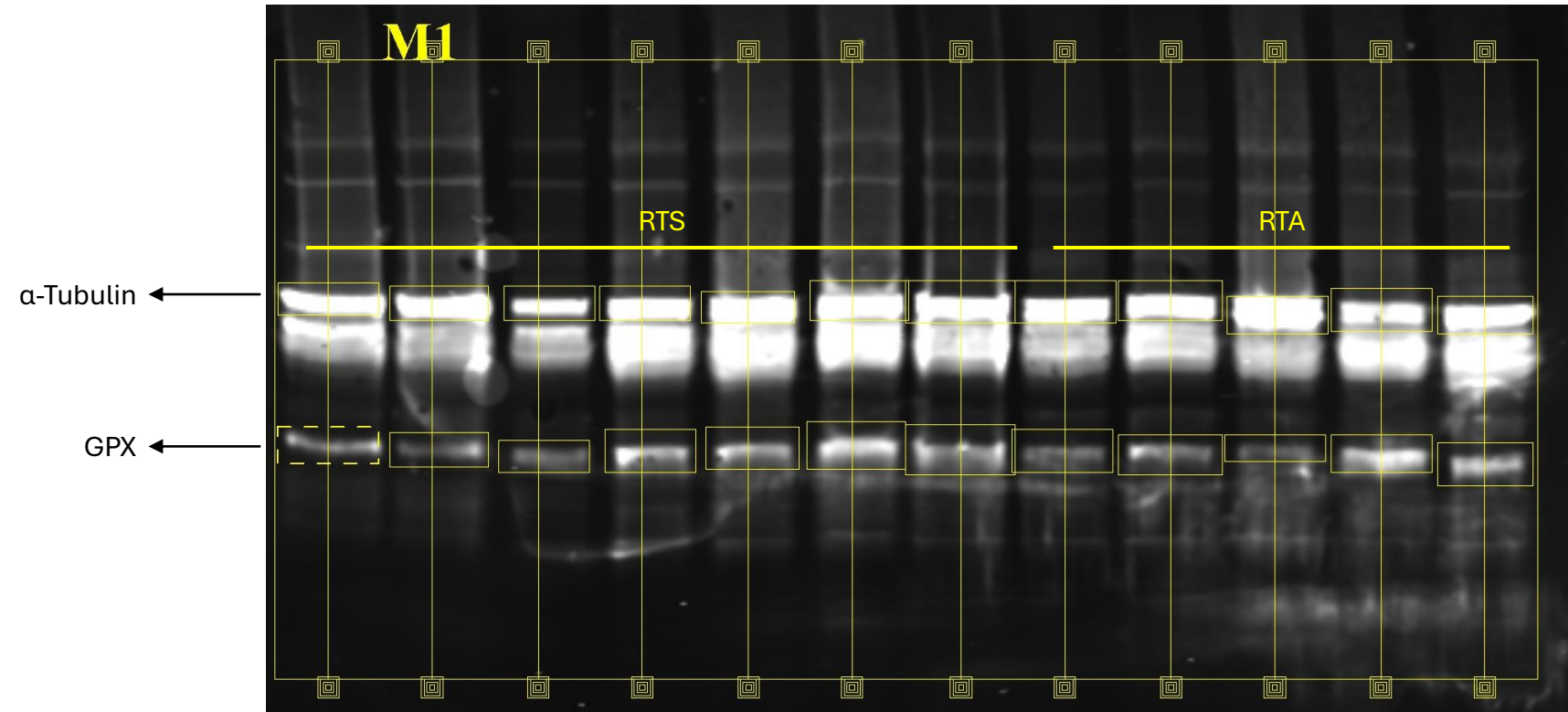

Supplement: Supplementary file 1 — Appendix S1. Western blot plots for all analyses assessed in the plantaris and soleus muscles. [file PHY2-13-e70334-s001.zip › PHYSREP-2025-02-130-T-f09-z-.pdf]

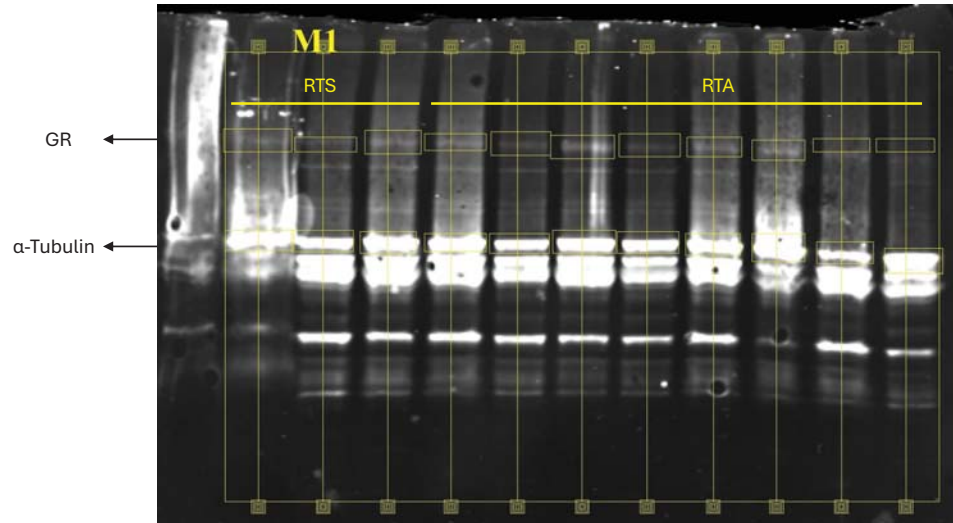

Supplement: Supplementary file 1 — Appendix S1. Western blot plots for all analyses assessed in the plantaris and soleus muscles. [file PHY2-13-e70334-s001.zip › PHYSREP-2025-02-130-T-f10-z-.pdf]

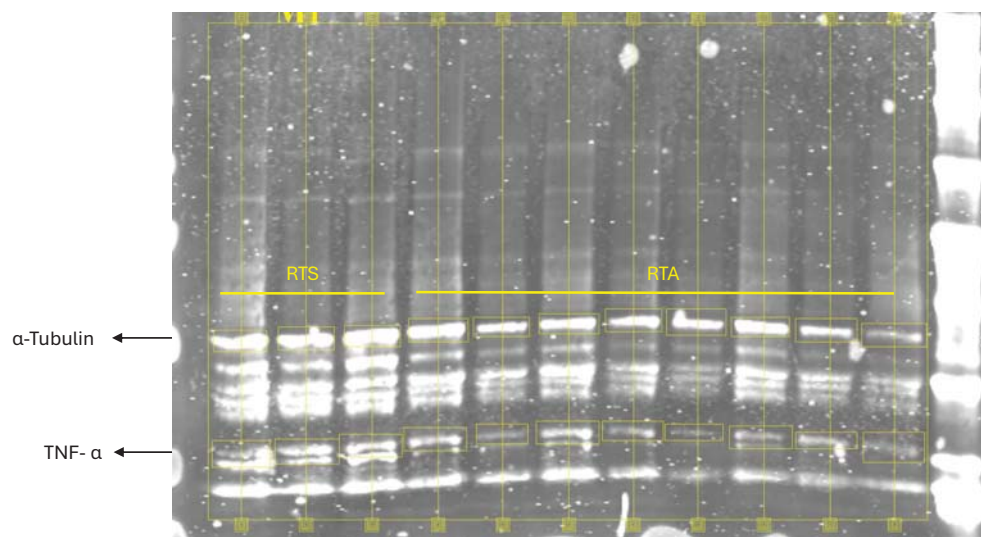

Supplement: Supplementary file 1 — Appendix S1. Western blot plots for all analyses assessed in the plantaris and soleus muscles. [file PHY2-13-e70334-s001.zip › PHYSREP-2025-02-130-T-f11-z-.pdf]

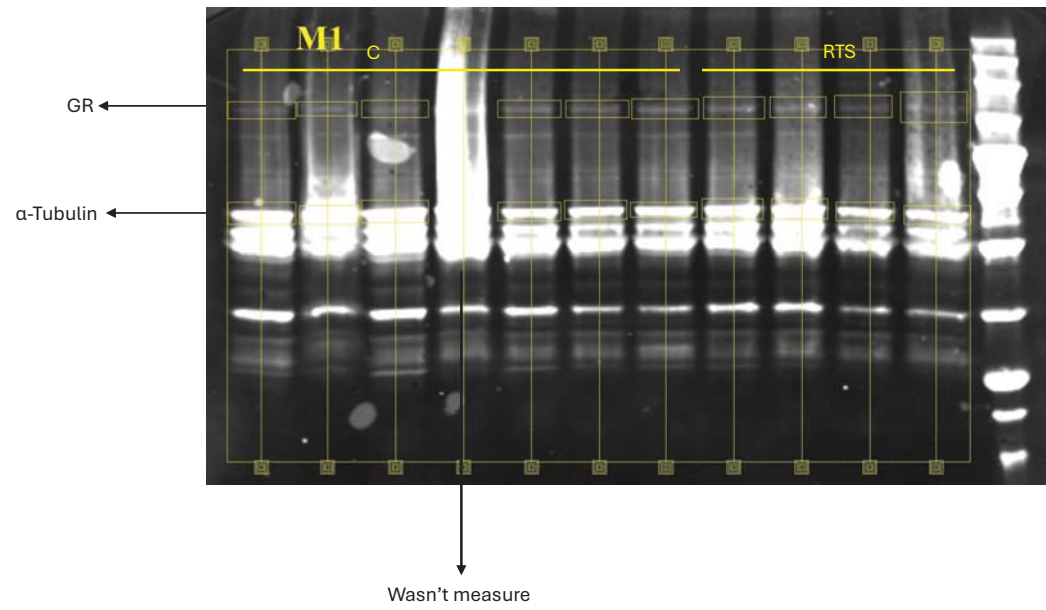

Supplement: Supplementary file 1 — Appendix S1. Western blot plots for all analyses assessed in the plantaris and soleus muscles. [file PHY2-13-e70334-s001.zip › PHYSREP-2025-02-130-T-f12-z-.pdf]

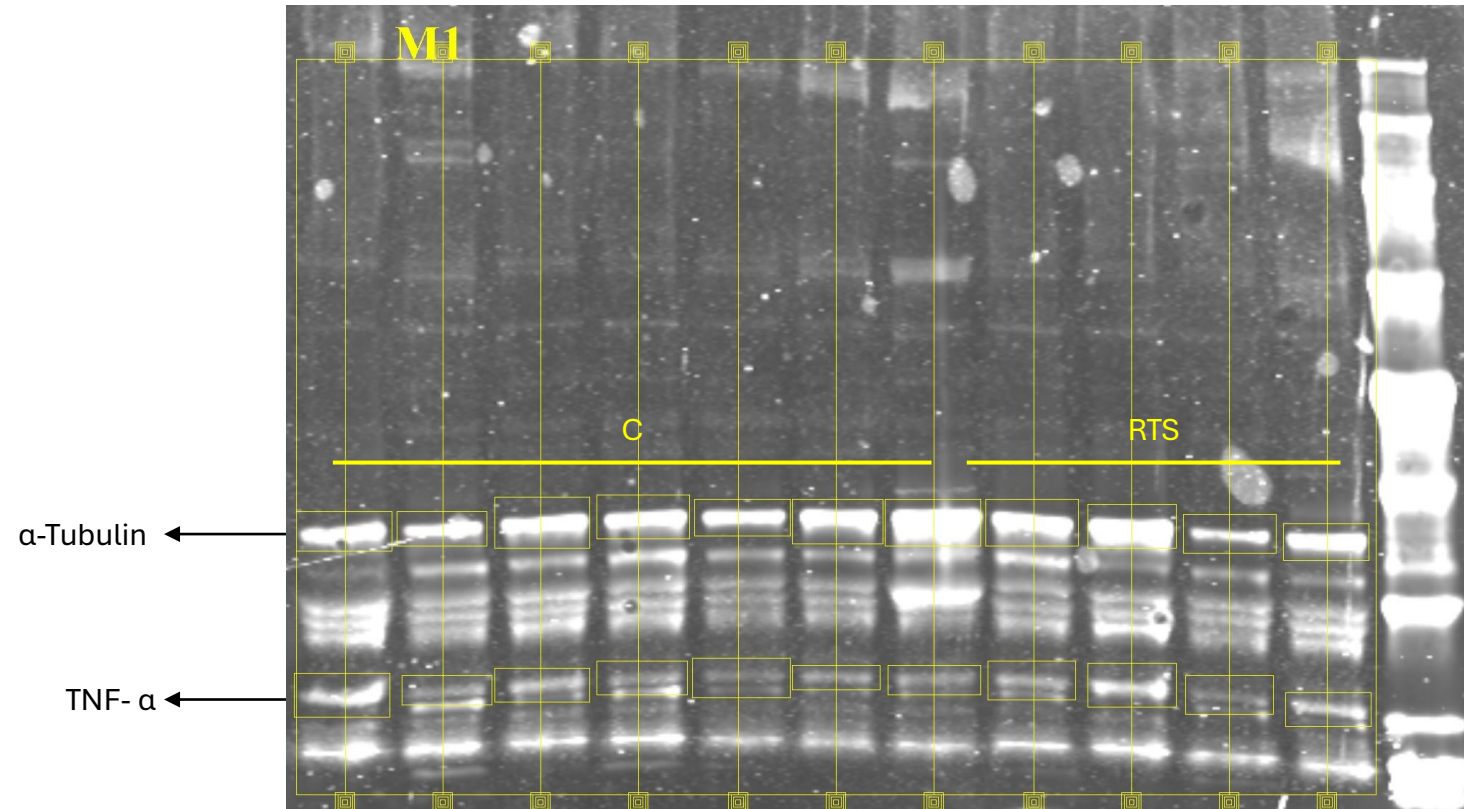

Supplement: Supplementary file 1 — Appendix S1. Western blot plots for all analyses assessed in the plantaris and soleus muscles. [file PHY2-13-e70334-s001.zip › PHYSREP-2025-02-130-T-f13-z-.pdf]

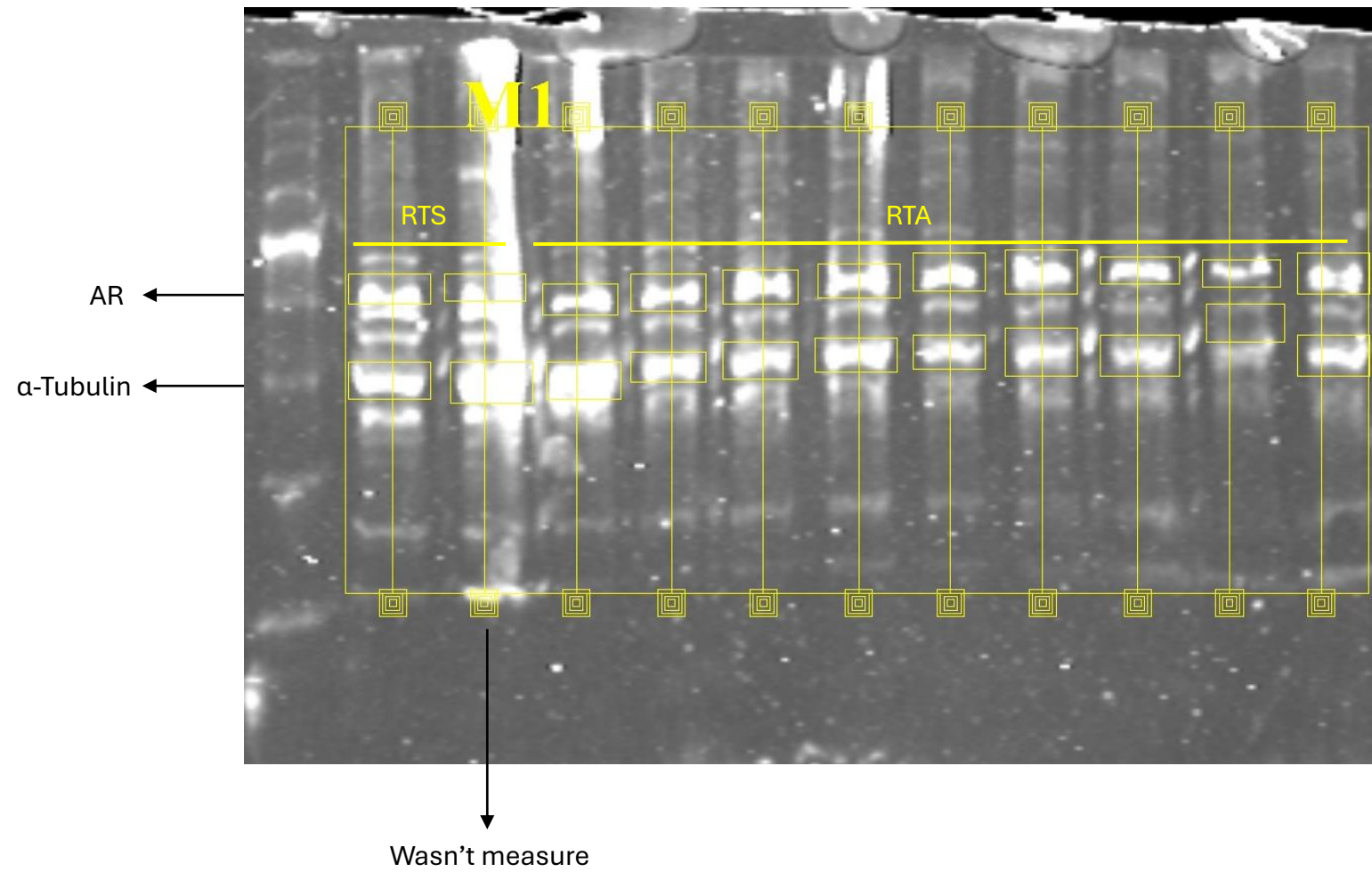

Supplement: Supplementary file 1 — Appendix S1. Western blot plots for all analyses assessed in the plantaris and soleus muscles. [file PHY2-13-e70334-s001.zip › PHYSREP-2025-02-130-T-f14-z-.pdf]

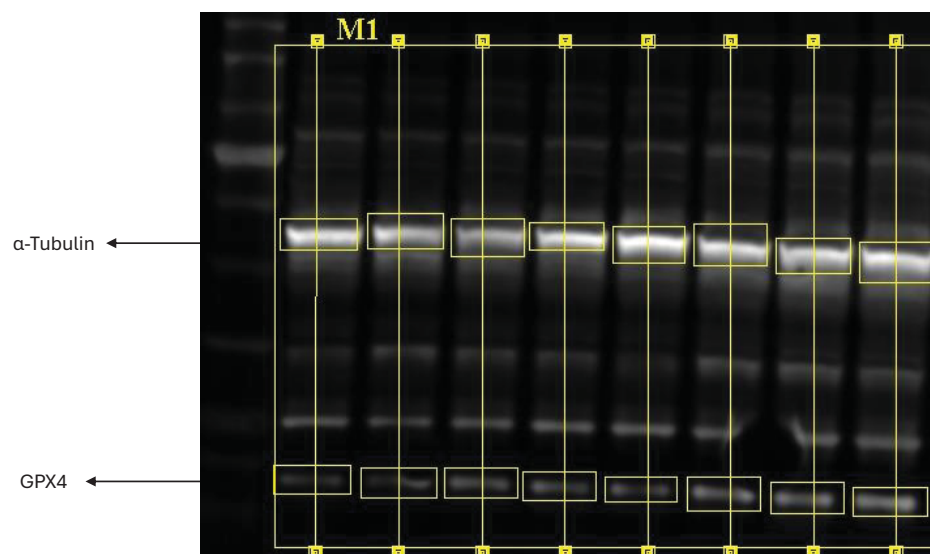

Supplement: Supplementary file 1 — Appendix S1. Western blot plots for all analyses assessed in the plantaris and soleus muscles. [file PHY2-13-e70334-s001.zip › PHYSREP-2025-02-130-T-f15-z-.pdf]

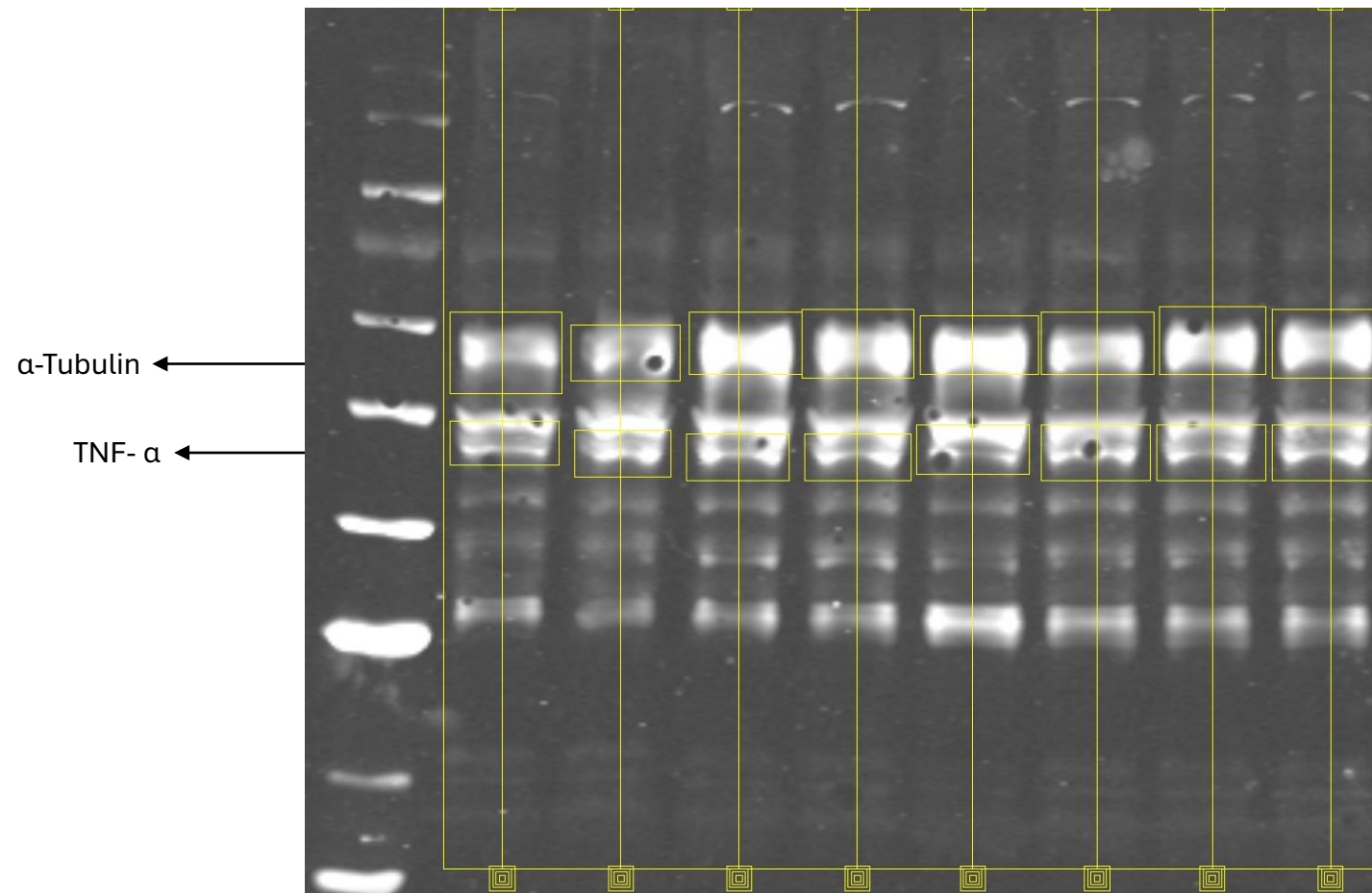

Supplement: Supplementary file 1 — Appendix S1. Western blot plots for all analyses assessed in the plantaris and soleus muscles. [file PHY2-13-e70334-s001.zip › PHYSREP-2025-02-130-T-f16-z-.pdf]

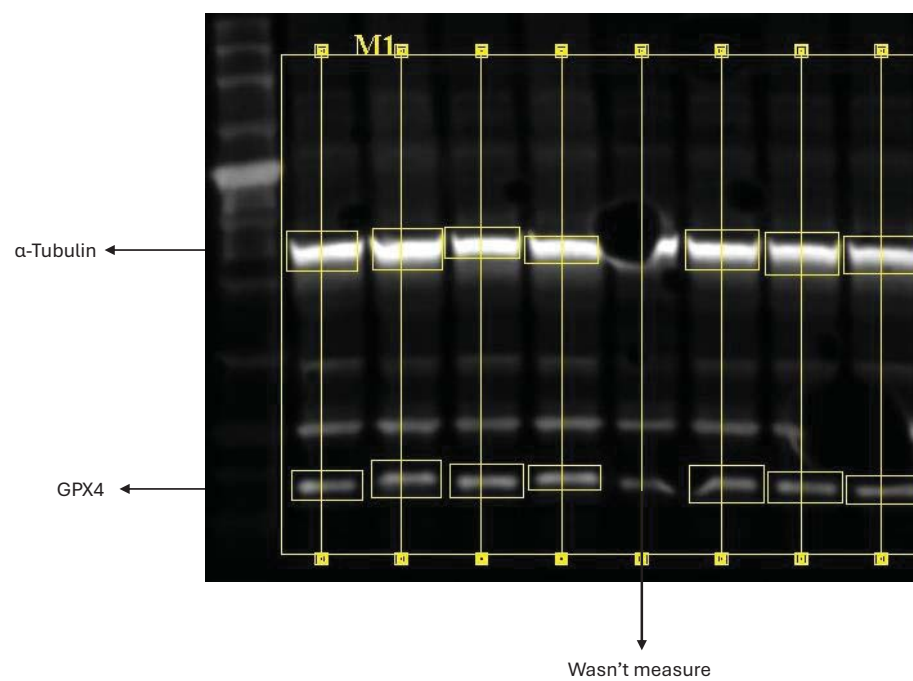

Supplement: Supplementary file 1 — Appendix S1. Western blot plots for all analyses assessed in the plantaris and soleus muscles. [file PHY2-13-e70334-s001.zip › PHYSREP-2025-02-130-T-f17-z-.pdf]

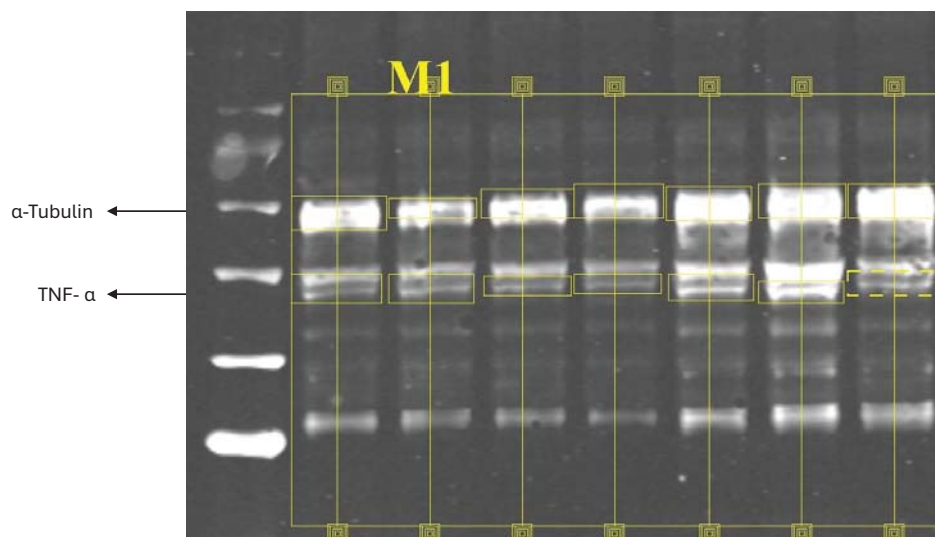

Supplement: Supplementary file 1 — Appendix S1. Western blot plots for all analyses assessed in the plantaris and soleus muscles. [file PHY2-13-e70334-s001.zip › PHYSREP-2025-02-130-T-f18-z-.pdf]

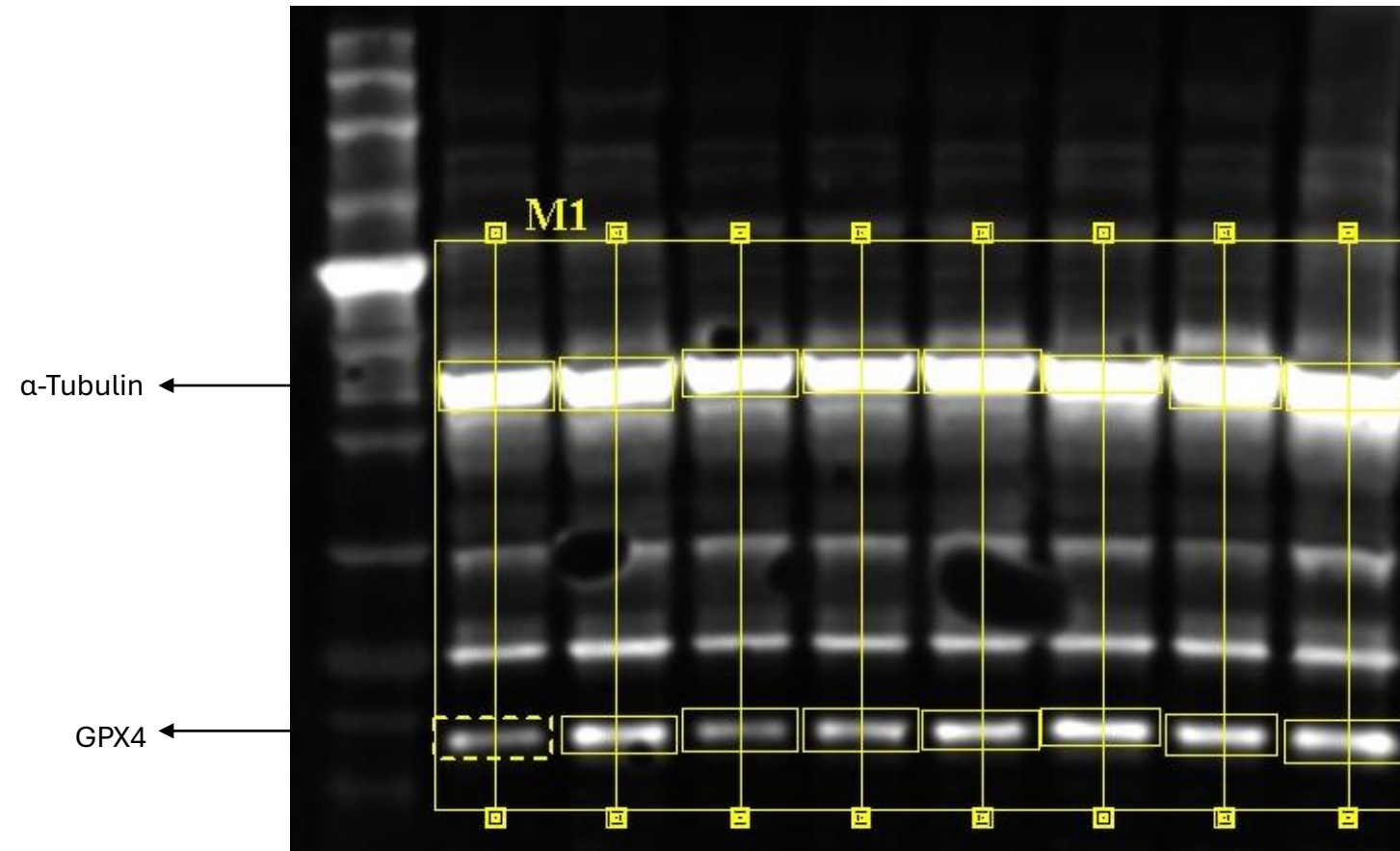

Supplement: Supplementary file 1 — Appendix S1. Western blot plots for all analyses assessed in the plantaris and soleus muscles. [file PHY2-13-e70334-s001.zip › PHYSREP-2025-02-130-T-f19-z-.pdf]

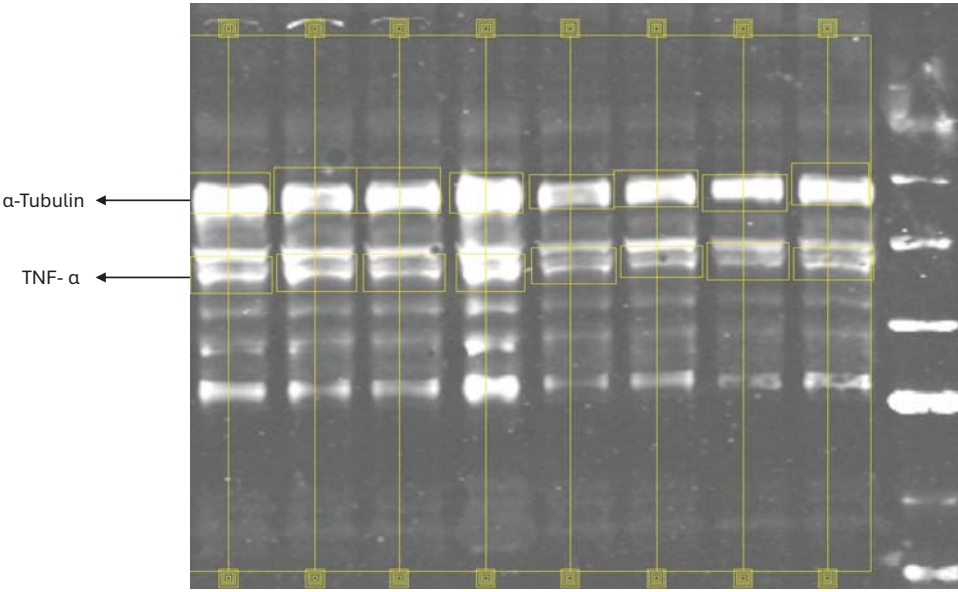

Supplement: Supplementary file 1 — Appendix S1. Western blot plots for all analyses assessed in the plantaris and soleus muscles. [file PHY2-13-e70334-s001.zip › PHYSREP-2025-02-130-T-f20-z-.pdf]

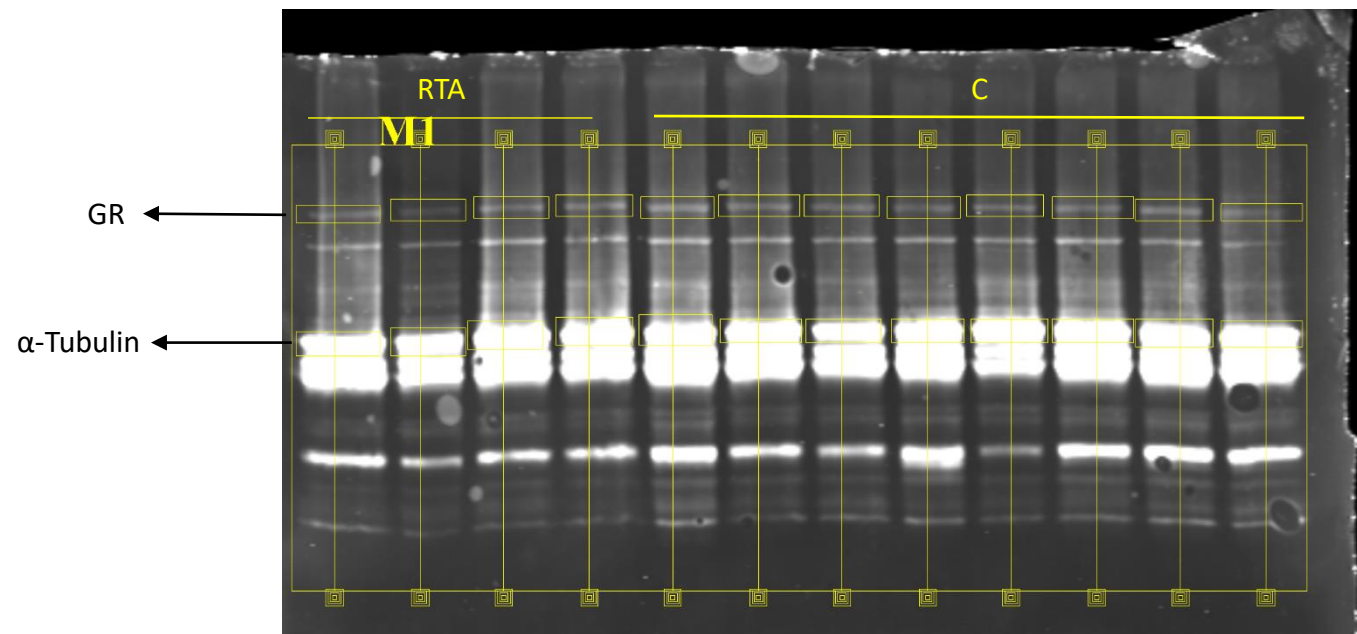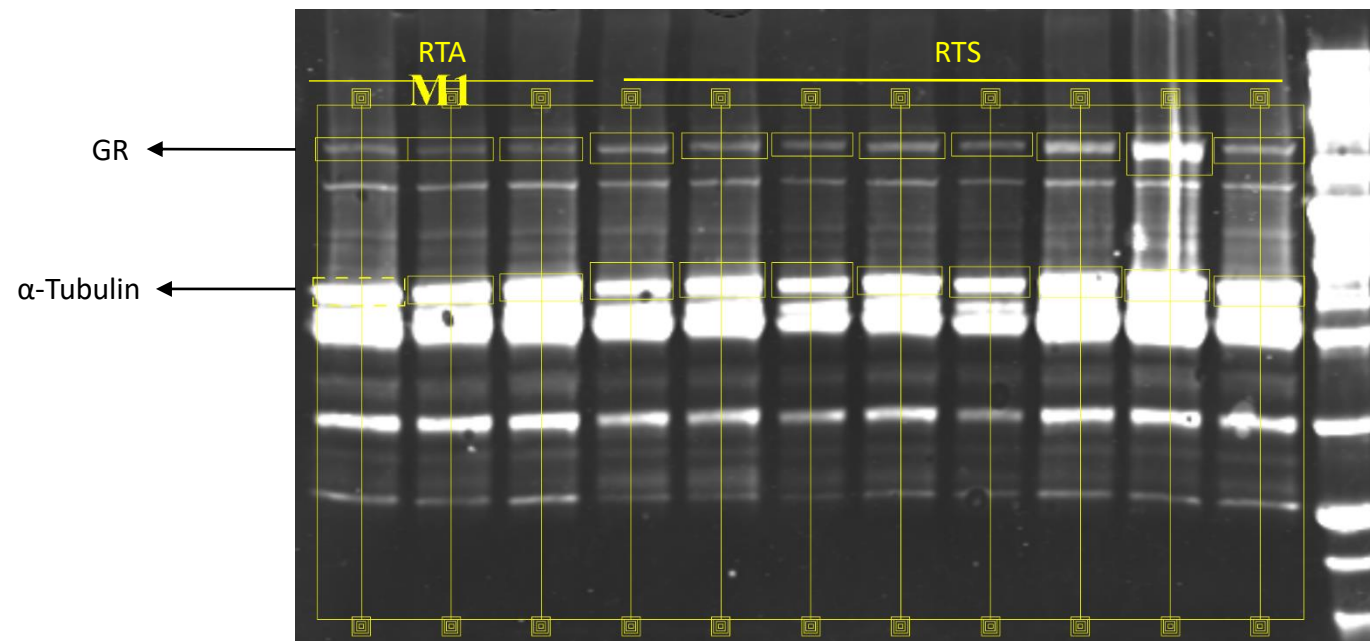

Supplement: Supplementary file 1 — Appendix S1. Western blot plots for all analyses assessed in the plantaris and soleus muscles. [file PHY2-13-e70334-s001.zip › PHYSREP-2025-02-130-T-f21-z-.pdf]
